# Effect of massage therapy on preterm neonate's body temperature

**DOI:** 10.4314/ahs.v21i3.44

**Published:** 2021-09

**Authors:** Emily Nyaga, Fabian Esamai, O'Brien Kyololo

**Affiliations:** 1 School of Nursing, Moi University; 2 School of Medicine, Moi University

**Keywords:** Body temperature, massage therapy, preterm neonates

## Abstract

**Background:**

Low-cost care strategies can be implemented to avert the morbidity and mortality associated with hypothermia in preterm neonates.

**Objective:**

To determine the effect of massage therapy on body temperature of preterm neonates.

**Methods:**

A quasi-experimental design was conducted among 72 preterm neonates at a level II special care nursery in Western Kenya. Neonates were recruited on the third day of life and followed up for 10 days. Neonates in the intervention group were massaged three times a day for 15 minutes. Body temperature was monitored and recorded before, during and after each therapy session. Neonates in the control group received routine care: temperature monitoring three times a day, feeding and diaper change.

**Results:**

Neonates who received massage had higher mean body temperature than the control group during therapy on day 6 (p = .019) and after therapy on day 6 (p = .017) and day 8 (p = .005). A comparison within massage group (before/during, during/after, before/after) showed an increase in mean body temperature during therapy compared to before therapy (p <.001) and after therapy compared to before therapy (p <.001).

**Conclusion:**

Massage therapy increases body temperature in preterm neonates.

## Introduction

Preterm neonates' ability to regulate body temperature is compromised owing to their large body surface area in relation to weight and relative lack of subcutaneous fat. Furthermore, the lack of facilities (e.g. incubators) compounds the preterm neonates' risk for hypothermia and cold stress^1^–^3^. Hypothermia results in variety of physiologic stresses including; increased oxygen consumption, metabolic acidosis, hypoglycemia, decreased cardiac output, and increased peripheral vascular resistance^3^. In order to avert these negative consequences of hypothermia, use of incubators has become a routine practice in neonatal units. Availability of the incubators is, however, a challenge in low-and middle-income countries (LMICs) such as Kenya.

Consequently, preterm neonates in LMICs are at higher risk for developing complications attendant with hypothermia such as poor feeding, apnea, and consequent morbidity and mortality^2^.To avert the deleterious consequences of hypothermia, nurses have adopted ingenious strategies such as kangaroo care^4^, ^5^. Studies in high-income countries have shown that other care strategies such as massage therapy, a moderate pressure stroking of preterm neonates, have the potential to increase preterm neonates' body temperature^6^. Researchers^7^–^9^ have attributed the increase in body temperature during massage therapy to a) therapists hands transferring heat to the neonate, b) facilitation of neurological temperature regulation by containment during message and c) increased blood circulation as a result of vasodilatation. It is, however, unclear whether similar effects of massage therapy on preterm neonates' body temperature would be witnessed in resource-limited settings such as Kenya. Therefore, this study aimed at determining the effect of massage therapy on body temperature of preterm neonates in an academic hospital in Kenya.

## Methods

A quasi-experimental study was carried out among 72 (36 each in massage and control

Nursery (SCN) in an academic hospital in the western region of Kenya. Neonates were recruited into the study if they a) were on breast milk or formula feeds via gavage or cup and spoon, b) were born at 28 to 37 weeks gestational age and c) weighed ≥1000grams based on significant neonatal mortality rate in neonates born before 28 weeks gestation and/or weighing <1000grams in MTRH^10^. Preterm neonates who were critically ill, those on ventilation support (continuous positive airway pressure [CPAP] or mechanical ventilation) and those with neonatal infections such as severe sepsis or necrotizing enterocolitis were excluded.

Consecutive sampling was used to recruit study participants. Neonates were recruited on day 3 of life to allow period for the researcher to explain to the mothers about the study. The first participant was recruited to the control group with the next neonate being recruited to the massage group. The recruitment continued in similar manner until the desired number for each group was attained. A study tool developed by the authors after thorough review of related literature was used to collect data on baseline characteristics including gestational age, birth weight and temperature at birth. Body temperature was also recorded before, during and after therapy in the massage group and thrice in 15 minutes for control group, all recordings were done three times a day for ten days. The study tool was reviewed for content validity by five pediatric nursing experts and suggestions made incorporated into the tool. Inter-rater reliability using two observers (research assistant and staff nurse at SCN) was done for temperature recording. Notably, there was no variation in temperature recording between the two observers.

In all the neonates, the massage therapy was performed by first author (EM). To be effective and not to cause harm (e.g. injury to internal organs) the first author was trained on the procedure by a specialized pediatric nurse with more than 10 years experience doing massage therapy. The training focused on the correct procedure of doing the massage with the main focus being ensuring that moderate pressure is applied on the body surface in a manner to cause effect and not harm the neonate. The training was conducted in three sessions each lasting two hours. The first and second session were conducted in the skills laboratory while the third session was carried out in a hospital setting.

The first session comprised review of massage procedure and watching the video clips. The second session was return demonstration on a dummy. The third session was practice on preterm neonates to achieve competency. The study intervention involved three massage sessions per day; morning (6 a.m.), afternoon (12 noon), and evening (6 p. m.) for 10 days starting day 3 of life. After thorough hand scrubbing, the researcher placed her warmed hands on the preterm neonate's body. Access ports were used for neonates in the incubator and the neonates in the cots were exposed only on areas being massaged. The rest of the body remained covered with blanket to prevent hypothermia. The massage therapy was given 1–2 hours after feeding to avoid discomfort and vomiting. A small amount of sunflower oil was used to prevent injurious friction between surfaces (providers' palms and neonate's skin) and was removed with cotton after the therapy. The therapy was temporarily stopped if the neonate started crying or passed urine or stool then continued when the neonate regained stability. The 15-minutes therapy included three standardized 5 minutes phases.

The phases were as follows:

**Phase 1:** Preterm neonates were placed in prone position. Moderate pressure (sufficient to produce slight skin indentation or slight skin color change from pink to white) was used to provide 12 strokes with palms of the hands, each stroke lasting 5 seconds. The strokes were provided in each area as follows: (a) head- from forehead hairline over scalp down to neck with alternate hands; (b) neck - from midline outwards with both hands simultaneously; (c) shoulders- from midline outwards with both hands simultaneously, and (d) back - from nape of neck down to buttocks, long stroke with alternate hands.

**Phase 2:** The preterm neonates were placed in supine position. Twelve moderate pressure strokes with palms of the hands, 5 seconds each, were provided in each area as follows: (a) forehead - from midline, outwards with both hands simultaneously; (b) cheeks - from side of nose, with both hands simultaneously in rotating and clockwise direction; (c) chest -'butterfly'stroking from midline upwards, outwards, downwards and inwards back to initiating point; (d) abdomen - from right iliac fossae, in a clock wise direction around abdomen avoiding the epigastrium and probes, with gentle strokes; (e) upper limbs (each separately) - from shoulders to wrist using alternate hands for stroking; (f) lower limbs (each separately) - from hips to ankles using alternate hands for stroking; (g) palms - from wrist to finger tips using alternate hands for stroking; and (h) soles - from heel to toe tips using alternate hands for stroking.

**Phase 3:** Joint stimulation was done for 5 minutes. The intervention comprised five passive flexion and extension movements of each large joint (shoulder, elbow, wrist, hip, knee, and ankle) for two seconds.

The massage therapy protocol was adopted from Mathai 2001^11^ a modification of Field et al. (1986) protocol for medically stable preterm neonates.

Neonates in the control group received the usual care which included routine body temperature monitoring three times daily, feeding and diaper change.

The body temperature reading of the neonates were recorded in degree Celsius to one decimal point before (baseline), during (after the 12 strokes), and after the massage using a temperature probe connected to a cardiorespiratory monitor (CODEC patient monitor CMS6000) attached to the chest. Neonates in the control group also had their temperature monitored for 15 minutes three times a day. The temperature probe was disinfected with spirit swab before and after each use to prevent infection. The RA's recorded the temperature reading from monitor to paper sheet (study tool), notably the RA's were not blinded to the allocation of the study participants since temperature recordings were done in the course of the intervention and thus, he/she could see what was being done on the neonate. Data were coded and enterd into Statistical Package for the Social Sciences (SPSS)version 20 database. Student t-test was used to (a) compare demographic characteristics between groups and (b) examine differences in body temperature between the groups. Paired t-test was employed to compare mean temperature within groups for before/during, during/after, and before/after. The study was registered under Clinical Trials.gov trial registration number NCT04287322 and approved by the Institutional Research & Ethics Committee (IREC) of the study hospital (approval number FAN: IREC 1573).

## Results

Out of the 72 singleton preterm neonates who were recruited, only 60 (30 in each group) completed the study; 10 participants (6 in treatment group and 4 in control group) were discharged before the 12^th^ day of life while 2 neonates in the control group died before completing the study. Neonates in both groups had similar body temperature at birth but the massage group had a lower gestational age (p = .032). (Table 1).

**Table 1:** Demographic Characteristics

Demographic Characteristics	Massage group	Control Group	p-value
Gestational age (weeks) Mean±SD	30.3±2.6	31.8±2.4	0.032*
Birth Weight (grams) Mean±SD	1273.7±167.8	1304.2±168.8	0.485
Temperature at birth Mean±SD	35.8±1.1	35.7±0.8	0.696

t: Student t-test

* significant at *p* ≤ 0.05

The lowest and highest mean body temperature was 35.2 and 36.5 respectively across the two groups. The mean temperature before therapy was higher in the control group than the massage group on days 4 (35.2±0.9 & 35.9±0.7; p = .002), 7 (35.9±0.5 & 36.4±36.4±0.9; p = 017), 8 (35.9±0.7 & 36.3±0.8; p = 0.035), 9 (35.7±0.6 & 36.2±0.7; p = .005), 10 (35.7±0.6 & 36.4±0.9; p = .003), 11 (35±0.7 & 36.4±0.7; p = .007), and 12 (35.9±0.6 & 36.4±0.6; p = .003).

The massage group had a higher mean body temperature than the control group during therapy on day 6 only (36.3±0.7 & 35.8±0.9; p = .019) and after therapy on days 6 (36.3±0.7 & 35.8±0.9; p = .017) and 8 (36.3±0.7 & 35.8±0.9; p = .005). (Table 2).

**Table 2:** Mean body temperature between groups findings

Day	Time	Massage	Control	Significance	
				t	p-value
3	Before stimulation	35.4±0.8	35.8±0.8	1.910	0.061
	During stimulation	35.9±0.7	35.8±0.8	0.787	0.434
	After stimulation	35.9±0.7	35.8±0.8	0.844	0.402

4	Before stimulation	35.2±0.9	35.9±0.7	3.323	0.002*
	During stimulation	35.7±0.9	35.9±0.7	0.945	0.349
	After stimulation	35.8±0.8	35.9±0.7	0.888	0.378

5	Before stimulation	35.6±0.8	36.0±0.9	1.874	0.066
	During stimulation	36.2±0.7	36.0±0.9	0.766	0.447
	After stimulation	36.2±0.7	36.0±0.9	0.667	0.507

6	Before stimulation	35.8±0.7	35.8±0.9	0.345	0.731
	During stimulation	36.3±0.7	35.8±0.9	2.419	0.019*
	After stimulation	36.3±0.7	35.8±0.9	2.460	0.017*

7	Before stimulation	35.9±0.5	36.4±0.9	2.472	0.017*
	During stimulation	36.3±0.5	36.4±0.9	0.191	0.849
	After stimulation	36.4±0.5	36.4±0.9	0.251	0.803

8	Before stimulation	35.9±0.7	36.3±0.8	2.157	0.035*
	During stimulation	36.5±0.6	36.3±0.8	1.046	0.300
	After stimulation	36.5±0.6	36.3±0.8	2.898	0.005*

9	Before stimulation	35.7±0.6	36.2±0.7	2.906	0.005*
	During stimulation	36.3±0.5	36.2±0.7	0.852	0.397
	After stimulation	36.3±0.4	36.2±0.7	0.887	0.379

10	Before stimulation	35.7±0.6	36.4±0.9	3.121	0.003*
	During stimulation	36.3±0.5	36.4±0.9	0.463	0.645
	After stimulation	36.3±0.4	36.4±0.9	0.560	0.577

11	Before stimulation	35.7±0.7	36.4±0.7	2.816	0.007*
	During stimulation	36.3±0.7	36.4±0.7	0.159	0.874
	After stimulation	36.3±0.6	36.4±0.7	0.222	0.825

12	Before stimulation	35.9±0.6	36.4±0.6	3.075	0.003*
	During stimulation	36.4±0.6	36.5±0.6	0.687	0.495
	After stimulation	36.5±0.5	36.4±0.6	0.659	0.513

Student t-test

* significant at *p* ≤ 0.05

Mean body temperature of the neonates did not differ within the control group; however, the temperatures were higher during than before therapy (p < .001) and after than before therapy (p < .001) in the massage group for the entire study period. (Table 3).

**Table 3:** Mean body temperature within group findings

Day	Significance within one group	Massage Group	Control Group
3	Before/during	0.0001*	-NA-
	During/after	0.570	-NA-
	Before/after	0.0001*	-NA-

4	Before/during	0.0001*	-NA-
	During/after	0.573	-NA-
	Before/after	0.0001*	-NA-

5	Before/during	0.0001*	-NA-
	During/after	0.579	-NA-
	Before/after	0.0001*	-NA-

6	Before/during	0.0001*	-NA-
	During/after	0.564	-NA-
	Before/after	0.0001*	-NA-

7	Before/during	0.0001*	-NA-
	During/after	0.647	-NA-
	Before/after	0.0001*	-NA-

8	Before/during	0.0001*	-NA-
	During/after	0.555	-NA-
	Before/after	0.0001*	-NA-

9	Before/during	0.0001*	-NA-
	During/after	0.923	-NA-
	Before/after	0.0001*	-NA-

10	Before/during	0.0001*	-NA-
	During/after	0.538	-NA-
	Before/after	0.0001*	-NA-

11	Before/during	0.0001*	-NA-
	During/after	0.639	-NA-
	Before/after	0.0001*	-NA-

12	Before/during	0.0001*	-NA-
	During/after	0.774	-NA-
	Before/after	0.0001*	-NA-

Paired t-test

* significant at *p* ≤ 0.05

-NA-: Not Applicable

## Discussion

To the best of our knowledge, this is the first study exploring the effects of massage therapy on preterm neonates' body temperature in a resource-limited setting. The study is of significance considering that majority of the preterm neonates in LMCs are nursed in cots.

Notably the control group had significantly higher temperature than massage group on certain days, possibly due to their relative higher GA with more mature physiological function capable of thermoregulation. Although the mean body temperature was significantly higher in the control than in the massage group at the beginning of therapy, it became significantly higher in massage than in the control group during and after therapy.

The mean body temperature remained constant in the control group during the 15-minutes observation period which is inconsistent with what has been reported elsewhere^6^.

Similar to previous studies^6^, ^12^, ^13^, we noted a significant increase in mean body temperature during massage therapy that remained elevated after therapy. Diego et al.^6^ found that preterm neonates' body temperature increased from baseline, reaching a peak during the massage and remained elevated during the post-massage period.

Inconsistent with current study findings, Mathai et al.^11^ didn't find a difference in temperature during massage therapy from the baseline recordings. Although the study has shed some light on the potential benefits of massage therapy on preterm neonates in low income settings, the findings should be interpreted with caution.

Firstly, the sample size was relatively small thus limiting the clinical significance of the findings. Furthermore, the broad definition of preterm neonates (28–37 weeks GA) in this study makes it impossible to tell weather the findings would apply to both very preterm (28–32 weeks) and moderate-to-late preterm (32–37 weeks) neonates.

Additionally, although the researcher and the RA's strived to maintain fidelity to the study, the fact that it was impossible to blind both to group allocation may have resulted in exaggerated rating of effects particularly with regard to body temperature reading for the intervention group.

## Conclusion

Massage therapy, a non-invasive, low-cost care strategy promotes body temperature regulation in preterm neonates that is maintained even after stimulation. Considering the dishearteningly high hypothermia-related neonatal morbidity and mortality particularly in preterm neonates in LMICs, there is need to (a) incorporate massage therapy as part of standard neonatal care and (b) train nurses and other health care providers on how to safely perform massage therapy on preterm neonates. Future studies should focus on effects of maternal massage therapy on preterm neonates' outcomes as a way of involving mothers in care of preterm neonates.
